# Contribution of the posterior parietal cortex in reaching, grasping, and using objects and tools

**DOI:** 10.3389/fpsyg.2014.00151

**Published:** 2014-03-05

**Authors:** Guy Vingerhoets

**Affiliations:** Department of Experimental Psychology, Ghent UniversityGhent, Belgium

**Keywords:** parietal cortex, reaching, grasping, tool use, intraparietal sulcus, inferior parietal lobule, dorsal stream, superior parietal lobule

## Abstract

Neuropsychological and neuroimaging data suggest a differential contribution of posterior parietal regions during the different components of a transitive gesture. Reaching requires the integration of object location and body position coordinates and reaching tasks elicit bilateral activation in different foci along the intraparietal sulcus. Grasping requires a visuomotor match between the object's shape and the hand's posture. Lesion studies and neuroimaging confirm the importance of the anterior part of the intraparietal sulcus for human grasping. Reaching and grasping reveal bilateral activation that is generally more prominent on the side contralateral to the hand used or the hemifield stimulated. Purposeful behavior with objects and tools can be assessed in a variety of ways, including actual use, pantomimed use, and pure imagery of manipulation. All tasks have been shown to elicit robust activation over the left parietal cortex in neuroimaging, but lesion studies have not always confirmed these findings. Compared to pantomimed or imagined gestures, actual object and tool use typically produces activation over the left primary somatosensory region. Neuroimaging studies on pantomiming or imagery of tool use in healthy volunteers revealed neural responses in possibly separate foci in the left supramarginal gyrus. In sum, the parietal contribution of reaching and grasping of objects seems to depend on a bilateral network of intraparietal foci that appear organized along gradients of sensory and effector preferences. Dorsal and medial parietal cortex appears to contribute to the online monitoring/adjusting of the ongoing prehensile action, whereas the functional use of objects and tools seems to involve the inferior lateral parietal cortex. This functional input reveals a clear left lateralized activation pattern that may be tuned to the integration of acquired knowledge in the planning and guidance of the transitive movement.

## INTRODUCTION

Despite the fact that genes encode an important deal of the information required by our motor system concerning locomotion, ingestion, and fight-and-flight responses, every individual must learn and remember a great deal of motor information during her or his lifetime. An important part of the human action repertoire that needs to be acquired consists of our remarkable ability to use a wide variety of objects as a means to achieve a diverse amount of goals. This unique quality of object-related (transitive) interaction is particularly developed in humans and involves the exposure to and learning of specific routines to master the correct gestures for functional object use, an ability called praxis.^1^ The neural basis of tool use is dramatically illustrated by the sudden deficits in the production of learned movements in patients suffering from apraxia following stroke. Tool perception and tool use have received a fair share of attention in recent functional neuroimaging research with paradigms ranging from visual tool perception to actual tool use. What all of these paradigms seem to have in common is that they elicit robust neural responses in areas of the posterior parietal, premotor, prefrontal, and posterior temporal cortices, and that this pattern of activation is clearly lateralized to the left hemisphere (Johnson-Frey, 2004; Lewis, 2006). The finding that this particular neural activation pattern is triggered by a diversity of tool-related tasks and stimuli underlines the importance of tools for our brain (and species) and also suggests that the neural network of operations underlying tool-related behavior is highly interconnected. The co-activation of distant neural regions during different types of tool-related tasks has obscured a detailed record of the functional role of each of these regions to tool use. In addition, the expanse of the neural response in the parietal, frontal, and temporal lobes has hampered the identification of a mosaic of specialized foci within each region as well as their specific contribution to transitive gestures.

Central to a functional transitive gesture are two other components of upper limb behavior that have been associated with a complex cortical organization, namely reaching and grasping. In contrast to the functional manipulation of objects, reaching, and grasping are readily observed in newborns and improve dramatically through practice within the first year of life. Much of the research on the neural correlates of reaching and grasping has been performed on non-human primates, but the emergence of neuroimaging has allowed a more fine-grained study in humans as well. Surprisingly, the scientific study on reaching and grasping and the research on object and tool manipulation have evolved as relatively independent fields with remarkably limited cross referencing in their literatures. Here, I will try to review the major observations on reaching, grasping, and the purposeful use of objects and tools. The focus is on the posterior parietal lobe and the action-related sub-regions within it, and how they contribute to goal-directed visuomotor action.

### ANATOMY OF THE POSTERIOR PARIETAL REGION

**FIGURE 1:**
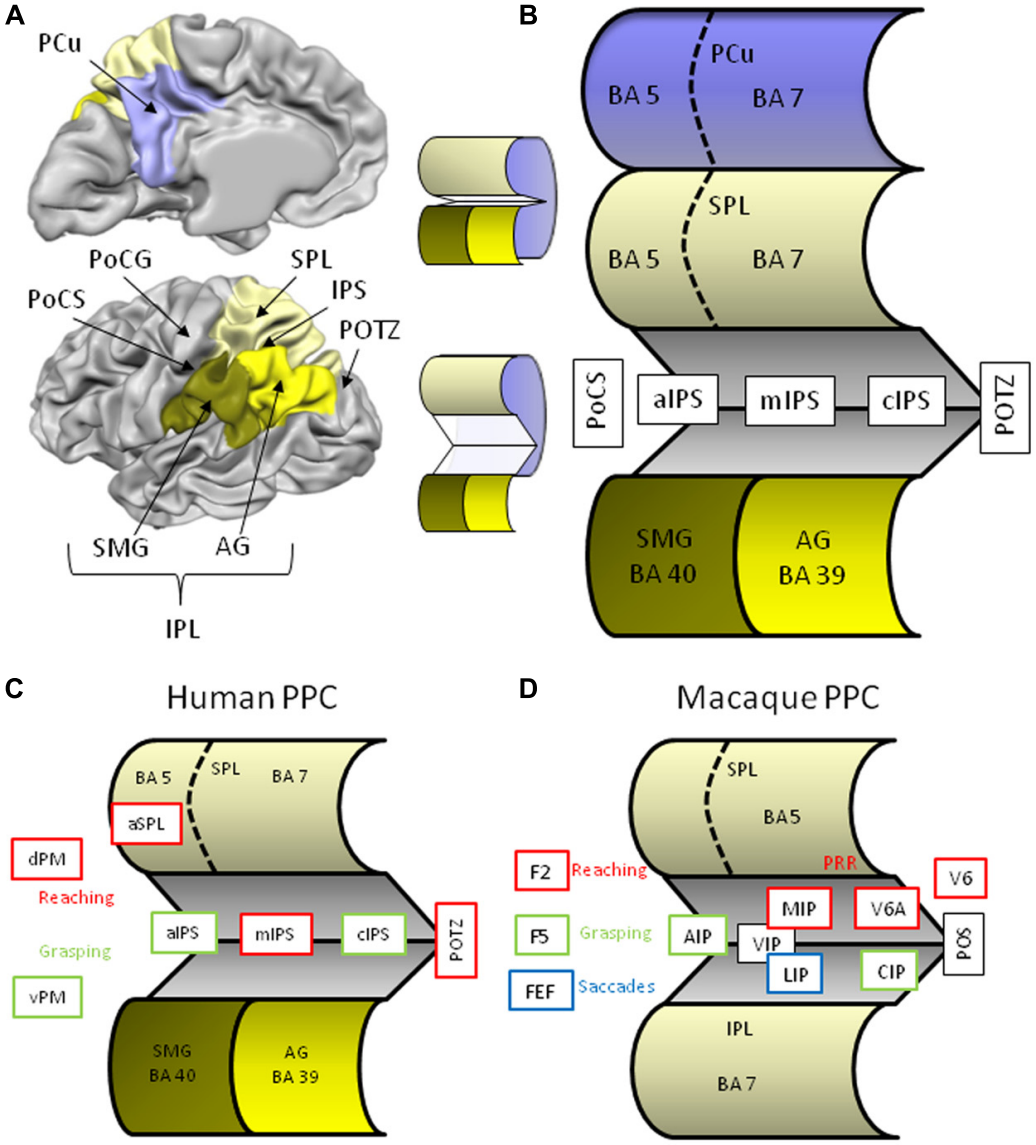
**(A)** Lateral and medial anatomical view of the left human hemisphere depicting the major parietal structures. Brain displays are adapted from snapshots of Brain Voyager’s Brain Tutor (http://www.brainvoyager.com/products/braintutor.html). **(B)** Schematic view of the human posterior parietal cortex with unfolded intraparietal sulcus. **(C)** Schematic view of the unfolded human posterior parietal cortex with regions indicating involvement in reaching (red) and grasping (green). **(D)** Schematic view of the unfolded macaque posterior parietal cortex with regions indicating involvement in reaching (red), grasping (green), and saccades (blue). Abbreviations in human brain: AG, angular gyrus; aIPS, anterior intraparietal sulcus; cIPS, caudal intraparietal sulcus; dPM, dorsal premotor cortex; IPL, inferior parietal lobule; IPS, intraparietal sulcus; mIPS, middle intraparietal sulcus; PCu, precuneus; PoCG, postcentral gyrus; PoCS, postcentral sulcus; POTZ, parieto-occipital transition zone; SMG, supramarginal gyrus; SPL, superior parietal lobule; vPM, ventral premotor cortex. Abbreviations in macaque brain: AIP, anterior intraparietal area; CIP, caudal intraparietal area; F2, frontal area 2; F5, frontal area 5; FEF, frontal eye fields; IPL, inferior parietal lobule; MIP, medial intraparietal area; LIP, lateral intraparietal area; POS, parieto-occipital sulcus; PRR, parietal reach region; SPL, superior parietal lobule; VIP, ventral intraparietal area; V6, visual area 6; V6A, visual area 6A.

Situated between the somatosensory cortex in the postcentral gyrus and the visual cortex in the occipital lobe, the posterior parietal cortex (PPC) is well positioned to bridge visual and somatosensory input and to contribute to the sensory control of action via output to the frontal (pre)motor areas. Anatomically, the lateral part of the PPC is divided in the superior and inferior parietal lobules separated by the intraparietal sulcus (IPS; **Figure 1A**). Anteriorly, the parietal lobules emerge out of the postcentral sulcus (PoCS) and posteriorly the small parieto-occipital sulcus (POS) forms the lateral boundary with the occipital lobe. The superior parietal lobule (SPL) consists of two cytoarchitectonically different regions, a smaller anterior Brodmann area (BA) 5, and a larger posterior BA area 7. These BA areas extend medially into the longitudinal fissure where they give rise to a similar division of the precuneus (PCu), the medial surface of the parietal lobe. The inferior parietal lobule (IPL) also consists of two different cytoarchitectonical regions that by and large correspond to two anatomical structures, namely the supramarginal gyrus (SMG) or BA 40, and posterior to it, the angular gyrus (AG) or BA 39. The IPS separating both lobules, is roughly about 4.5 cm long and ascends anteriorly from the postcentral sulcus (aIPS), runs a horizontal course over its middle segment (mIPS), and then descends caudally [segment called caudal IPS (cIPS)] where at its most posterior end (pIPS) merges with the POS. The IPS is quite deep, in some regions up to more than 3 cm, and a lateral (sometimes called horizontal) and medial bank are distinguished. In order to expose these intraparietal regions that remain concealed in a classical lateral view of the brain I have constructed a schematic image of the PPC (**Figure 1B**).

Although our knowledge on the sensory control of action has benefitted a lot from macaque neurophysiology, anatomical and functional homologies of the primate brain in humans are highly tentative. First, the monkey’s parietal lobe is cytoarchitectonically quite different from ours. Macaques do not have BA’s 39 and 40, rather their IPL is made up of (subdivided) BA 7, whereas their SPL is BA 5 (**Figure 1D**). In addition, their IPS consists of many specialized regions that appear to be organized differently in human IPS. Second, compared to non-human primates, the magnitude and complexity of human tool use reflects a profound discontinuity between us and our close relatives with regard to the cognitive capacities underlying tool use (Vaesen, 2012). As a consequence of these important differences between species, we will focus on findings from human neuropsychology and neuroimaging, although we will refer to monkey research when it comes to more basic components of transitive gestures such as reaching and grasping.

## REACHING

### DEFINITION

Reaching can be described as the transportation of the hand to the object by the upper limb^2^. Obviously, this requires an integration of the hand and target positions into a single reference frame, thus combining proprioceptive and visual information. A detailed review of the vast literature on the sensorimotor integration of eye-hand coordination, gaze modulation, and (near) space coding is beyond the scope of this contribution (Carey et al., 2002; Culham et al., 2008). Instead, we will focus on the parietal correlates of simple reaching tasks in humans.

### NEUROPSYCHOLOGICAL RESEARCH

The classical deficit associated with difficulties in reaching is optic ataxia (OA). Although these patients typically do not exhibit problems when reaching for objects in central vision and show no signs of motor or sensory disturbances, neglect, or apraxia, they are severely impaired when reaching to targets in the peripheral visual field (Perenin and Vighetto, 1983, 1988; Prado et al., 2005). Patients with OA present with reaching errors of their contralesional hand in both visual fields, and also on the presentation of the object in the contralesional hemifield. This hand and field effect acknowledges the problems of visuomotor integration in OA. Recent data of a patient with a selective lesion in left PPC demonstrates the function specificity of OA, showing impairment of reaching (but not grasping) and effector independence (disturbances of arm *and* leg reaches; Cavina-Pratesi et al., 2013). Common lesion sites of patients with OA include the IPS and SPL. Voxel-based lesion function mapping later contradicted the involvement of the SPL proper, and pointed to the parieto-occipital transition zone spanning the IPL, SPL, and PCu border with the superior occipital cortex instead (Karnath and Perenin, 2005).

### NEUROIMAGING RESEARCH

Because of the difficulty of studying arm and hand movements within the scanner bore, researchers have turned to different solutions for the viewing and motor limitations of the MR-setting. Instead of true reaching, some studies asked participants to orient the wrist and point with the index finger, thus eliminating the transport component of the reach movement. In addition, target presentation during fMRI is classically achieved by means of back projection through a mirror fixed to the head coil. This arrangement, however, induces a discrepancy between the spatial reference frame of the movement and that of the target, and mirror conditions have been shown to impact neural activations patterns and even behavior (Binkofski et al., 2003). Solutions have been proposed to offer direct viewing of the target that allow for more natural reaching responses (Prado et al., 2005; Beurze et al., 2007).

**FIGURE 2:**
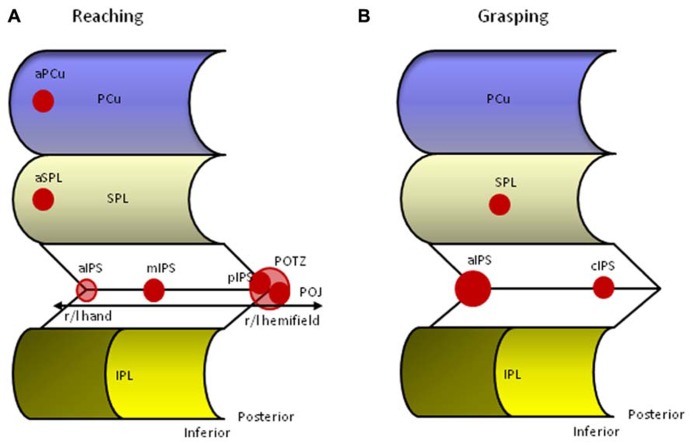
**Human parietal foci responsive to reaching (A) and grasping (B).** aIPS, anterior intraparietal sulcus; aPCu, anterior precuneus; aSPL, anterior superior parietal lobule; cIPS, caudal intraparietal sulcus; IPL, inferior parietal lobule; mIPS, middle intraparietal sulcus; PCu, precuneus; pIPS, posterior intraparietal sulcus; POJ, parieto-occipital junction; POTZ, parieto-occipital transition zone; SPL, superior parietal lobule.

Blangero et al. (2009) performed a meta-analysis on neuroimaging studies of reaching to identify the relevant parietal foci and to compare these foci with lesion studies from OA patients. Using an activation likelihood estimation (ALE) method on thirteen empirical studies they found four bilateral parietal foci along an antero-posterior axis involved in reaching (**Figure 2A**). The most posterior pair is located on the inferior side of the POS, a location also referred to as the parieto-occipital junction (POJ). The second pair is located on the opposite (superior) bank of the POS in posterior IPS (pIPS). The third pair is situated in mIPS, and finally, the last pair is located in aIPS, a region that is often associated with grasping. It is of importance to note that reaching generally elicits bilateral activation along the IPS, but that the hemisphere contralateral to the moving hand is substantially more involved than the ipsilateral hemisphere. In addition, Blangero et al. (2009) were able to show that the more posterior foci (pIPS and POJ) displayed greater lateralization for contralateral visual stimulation, whereas the more anterior foci (aIPS and mIPS) revealed higher lateralization for the use of the contralateral hand, thereby demonstrating an antero-posterior gradient of these modules to somatic-to-visual integration (Vesia et al., 2010; Vesia and Crawford, 2012). Finally, lesion overlap of 11 OA patients superimposed on the parietal clusters derived from the meta-analysis showed overlap with the three most posterior foci (Blangero et al., 2009).

It has proven difficult to disentangle the functional contribution of each of these regions to reaching. A popular option is to compare the functional organization of monkey IPS to that of humans (Culham and Kanwisher, 2001; Grefkes and Fink, 2005; Culham et al., 2006; Vesia and Crawford, 2012). Electrophysiological and anatomical research in the macaque revealed a mosaic of interconnected IPS areas that encode combinations of sensory and effector information that define their functions. Roughly, anterior parts are involved with sensorimotor processing and posterior parts with visual processing. In addition, neurons in the medial bank respond more to arm movements, and neurons in the lateral bank are more concerned with eye movements. Neurophysiological testing of IPS neurons according to their preferences resulted in the differentiation of several functional regions named after their anatomical location in the anterior (AIP), middle (MIP, LIP), fundus (VIP), and posterior (CIP, V6 complex) portions of the monkey IPS (**Figure 1D**). Two of these areas seem of particular importance for reaching, the V6 complex and MIP. The monkey V6 complex consists of a purely visual V6 area that receives input from early visual areas and sends output to the V6A visuo-motor area and MIP. Together with MIP, the V6 complex forms the monkey parietal reach region (PRR) that makes a circuit with macaque area F2, a dorsal premotor region located anterior to primary motor cortex. Similar to the monkey, human pIPS and POJ are on each side of the POS, making this area a likely candidate for the putative human V6 complex (Pitzalis et al., 2013). A recent series of experiments confirmed the specific contribution of the human superior parieto-occipital cortex (SPOC) for visually guided reaching (Quinlan and Culham, 2007; Culham et al., 2008; Filimon et al., 2009; Gallivan et al., 2009; Cavina-Pratesi et al., 2010).

Neuronal discharge in the macaque MIP is dependent on the direction of hand movements toward a visual target and appears involved in the coordination of hand movements and visual targets (Johnson et al., 1996; Eskandar and Assad, 1999; Grefkes and Fink, 2005). In humans, true reaching tasks also show increased neural activity in mIPS and this region is deemed crucial for transforming visual coordinates into motor programs (Grefkes et al., 2004; Grefkes and Fink, 2005; Prado et al., 2005). The association between visual and motor coordinates is in line with the observation that the mIPS is also robustly activated in paradigms requiring visually guided saccadic eye movements and may be involved in the planning of eye movements in relation to the goal that is to be achieved (Beurze et al., 2009; Filimon et al., 2009; Vingerhoets et al., 2010).

Finally, the anterior bilateral IPS pair reported in the Blangero et al. (2009) overview can be associated with monkey AIP. Situated on the lateral bank, monkey AIP contains neurons that are highly selective to the size, shape, and orientation of objects, and are active during fixation and manipulation of objects (Grefkes and Fink, 2005; Culham et al., 2006). Although aIPS is also activated during reaching, it is more active during grasping, and we will discuss the AIP-aIPS comparison in more detail below (Culham et al., 2003).

Whereas most reaching related parietal activation appears to be located bilaterally along the IPS, recent studies have documented activation of superior regions within the PPC. Filimon et al. (2009) and Filimon (2010) reported a reach selective area in the anterior precuneus (aPCu). This medial parietal region was equally active in a reaching-to-target task with the hand visible as in a similar (darkened) task in which the participant could not see his/her hand, suggesting that aPCu is a sensorimotor region whose sensory input is primarily proprioceptive. This finding underlines the observation that there may be multiple reach-related areas within the PPC with greater visual dominance in posterior parts, mixed responses in between, and greater or even exclusive somatosensory dominance in anterior PPC regions (Filimon, 2010). Cavina-Pratesi et al. (2010) reported, besides the already mentioned SPOC, also activation in the rostral SPL during a task that manipulated the transport component by positioning the target in a far or near location. A recent neuroimaging study that directly compared reaching and grasping movements reported a functional gradient of specificity with grasp-specific regions being located in the left anterior IPS extending in the PoCS, regions along the IPS showing activation during reaching *and* grasping movements, and reach- specific activation in the left PCu and SPL (Konen et al., 2013). Together, these data suggest that in comparison to tasks involving object grasping or manipulation (see below), reaching without grasping activates more dorsal and medial parts of the PPC (Filimon et al., 2007; Cavina-Pratesi et al., 2010; Konen et al., 2013).

### OBSERVATION AND IMAGERY OF REACHING

Only few studies investigated the observation and/or mental simulation of reaching movements. Compared to passive viewing of objects (baseline), observed reaching and imagined reaching activated the IPS, SPL, and PCu (Filimon et al., 2007). Imagined reaching also included SMG activation. In both conditions left hemispheric activations were much stronger as these right handed volunteers observed/imaged the reaching tasks with the right hand. Significant overlap between activations during executed, observed and imagined reaching was found in the left medial IPS and the left SPL extending medially into the superior PCu. The contrast between observed and imagined reaching showed no difference in parietal activation suggesting an equal neural response in observation and imagery of reaching (Filimon et al., 2007).

## GRASPING

### DEFINITION

Whereas reaching requires correspondence between the spatial location of hand and target, grasping is focused on another visuomotor match, namely the correspondence between the object’s form and the hand’s posture. Grasping requires the extraction of visual features of an object, such as its size, shape, orientation, texture, and estimated weight in order to properly preshape the hand during the approach, adjust grasping speed during contact, and close the fingers around the object applying the correct grip force. Although reaching and grasping can be distinguished conceptually, in practice they form a continuum as revealed by the kinematics of a reach-and-grasp movement showing adaptation of the grip aperture during the reaching phase of the gesture (Jeannerod et al., 1995; Castiello, 2005). The close relation between the transport and grip components of prehension make it difficult to separate the neural correlates underlying each component. As a result, several areas are activated during reaching *and* grasping, although often a preference in responsiveness to reaching or grasping can be observed.

### NEUROPSYCHOLOGICAL RESEARCH

Binkofski et al. (1998) performed a lesion analysis in nine patients with parietal lesions who showed no or only minor visuomotor difficulties and underwent kinematic analysis during reach-and-grasp movements. Patients showing kinematic deficits (*n* = 5) all revealed lesions in the lateral bank of the aIPS, whereas in patients showing normal grasping this region was spared. Kinematics further revealed that the deficit was more pronounced for grasping than for reaching, and especially affected the contralesional hand. To the best of my knowledge, this is the only study that investigated grasping in a clinical population with parietal lesions.

### NEUROIMAGING RESEARCH

Similar to reaching, the technical limitations of the scanner have influenced the ecological validity of the grasping paradigms used. As grasping involves objects *and* movement – two well known sources of artifacts during MR-data acquisition – researchers have used pantomimed grasping (no object) or imagined grasping (no object, no movement) instead. Again, these are rather unnatural, or at least uncommon, tasks that question the validity of these paradigms’ claims on the neural representation underlying real grasping. Fortunately, methodological and technical solutions have been presented over the last few years that allow a more natural setup within the scanner environment (Culham et al., 2006).

Data from neuroimaging studies have confirmed the importance of the junction between the PoCS and the aIPS for human grasping (**Figure 2B**; Grafton et al., 1996b; Culham et al., 2003; Shikata et al., 2003; Frey et al., 2005; Pierno et al., 2009). The cortex in this region is considered to be part of the IPL. As described above, the region also responds to reaching movements, but its response to grasping is generally stronger (Culham et al., 2003, 2006). Similar to non-human primates, the aIPS is activated by visually guided grasping, object manipulation without vision, and visual inspection without grasping (Culham et al., 2006). The latter effect is only achieved when 3D objects are presented or when 2D pictures of objects with particular hand associations are shown, such as tools (Chao and Martin, 2000; Culham et al., 2003; Creem-Regehr and Lee, 2005). Pure perceptual processing of object features unrelated to grasping does not activate aIPS (Culham et al., 2003). Grasping with either hand evokes bilateral aIPS activity, but the extent and magnitude of the activation is much larger in the aIPS contralateral to the hand used and appears influenced by handedness (Culham and Kanwisher, 2001; Culham et al., 2003; Begliomini et al., 2008). Finally, TMS applied to the left aIPS (but not mIPS or cIPS) disrupts on-line grasping execution (Rice et al., 2006), and selectively results in impaired judgments of tool-related grip configurations in right handers (Andres et al., 2013). World-wide replications of anterior IPS activation during grasping paradigms in humans and macaques result in a growing consensus that human aIPS is the most likely functional equivalent of monkey AIP. In humans, the role of the aIPS region has also been extended to higher-order motor functions as it appeared involved with action planning, recognition of goal-directed hand-object movements, and motor semantics (Shmuelof and Zohary, 2005; Tunik et al., 2005, 2007, 2008b; Hamilton and Grafton, 2006; Ortigue et al., 2009; Vingerhoets et al., 2009a; Cross et al., 2012).

Macaque AIP forms a circuit with macaque F5, the rostral part of the monkey ventral premotor cortex (vPM) which, in turn, projects to the hand region of the primary cortex F1 (Jeannerod et al., 1995). Inactivation of either the monkey AIP or F5 area gives rise to impaired hand shaping relative to the object’s features (Gallese et al., 1994; Fogassi et al., 2001). It was suggested that AIP uses visual input to highlight grasp-relevant object features and that F5 uses this information to select the most appropriate grasp. Continuous feedback between both regions monitors the ensuing grasp movement (Fagg and Arbib, 1998). A similar fronto-parietal link has been proposed in humans, linking aIPS with the putative human homologue of monkey F5, the pars opercularis, the posterior part of the inferior frontal gyrus, also known as the vPM or Broca’s region (**Figure 1C**). Recent neuroimaging studies have corroborated this idea (Tunik et al., 2005; Cavina-Pratesi et al., 2010; Gallivan et al., 2011; Makuuchi et al., 2012; Vingerhoets et al., 2013b). As in the monkey F5 region, vPM in humans is modulated by grip type, in particular precision grips (Ehrsson et al., 2000, 2001). But also aIPS shows selective responses to different hand configurations. Multivariate pattern classification analysis of BOLD responses during a rock-paper-scissors game was able to accurately classify the pattern of aIPS activity unique to each hand movement (Dinstein et al., 2008). Accurate classification was obtained within modality (either during observation or execution), but not between modalities, leading the authors to suggest that observed and executed movements may be represented by different subpopulations of neurons within aIPS (Dinstein et al., 2008). Although this study investigated hand movements (postures) rather than grasps, these results disclose the central role of aIPS in the perception and execution of hand configurations.

Grasping also elicits activation in other parietal regions besides aIPS. Activation during visually guided grasping was reported in the posterior section of IPS (Culham et al., 2003). In order to explain the pIPS activation during grasping, another comparison with the monkey brain appears relevant. Macaque CIP is situated in the lateral bank of caudal IPS and appears involved in the analysis of object features such as surface texture and orientation (**Figure 1D**). It is believed to analyze the 3D shape and orientation of objects by integrating binocular and monocular depth cues and feed this to the grasping area AIP (Sakata et al., 1998; Tsutsui et al., 2003; Grefkes and Fink, 2005). In humans, caudal activation in the medial bank of IPS was uncovered in a surface orientation discrimination task (Faillenot et al., 1999; Shikata et al., 2001, 2003).

A second parietal region linked with aIPS during grasping lies in SPL (Tunik et al., 2008b). Tunik et al. (2008b) had right handed participants grasp target objects that could or could not undergo rotation after the initiation of the reach and grasp movement. Electrophysiological recordings of evoked brain responses revealed a two-stage process. Response duration in a first stage activated left aIPS region and was longer when there was an object perturbation, whereas initiation of the corrective movement coincided with SPL activity. The authors suggested that aIPS is involved in the initial state activation and the emerging action plan. With increasing discrepancy between the desired and actual state, aIPS activation is prolonged to initiate corrections that are mediated in part by the SPL (Tunik et al., 2008b).

### OBSERVATION AND IMAGERY OF GRASPING

Observed and imagined grasping actions have been studied frequently with neuroimaging as they require no actual movements in the scanner. Based on the temporal coupling between executed and imagined movements a similarity, in neural terms, was expected between the state where an action is simulated and the state of execution of that action (Jeannerod, 2001). In monkeys an extensive overlap of parietal networks activated during grasp execution and grasp observation have been established (Evangeliou et al., 2009). Most studies on imagined grasping in humans indeed reported similar activation of the IPS, SPL, and IPL areas compared to executed grasping (Decety et al., 1994; Grafton et al., 1996a; Binkofski et al., 1999; Grezes and Decety, 2002). Also the observation of grasping actions is believed to elicit the same mechanisms in the observer’s brain that would be activated were that action intended by the observer. This prediction was confirmed by several neuroimaging studies that compared observed versus executed object grasping (Grafton et al., 1996a; Buccino et al., 2001; Grezes et al., 2003a). A recent study required volunteers to judge videos of transitive reach and grasp gestures and decide whether the object was grasped with the intention to use or to displace. Discrimination of action intention during observed grasping revealed bilateral activation of aIPS, mIPS, and cIPS foci suggesting that regions very similar to those involved with executed grasping are recruited by the observer to determine the purpose of the grasp (Vingerhoets et al., 2010). Lateralization of the posterior parietal activation during observed grasping, in particular of the aIPS, appears influenced by the observer’s perspective. In a first-person perspective, anatomical congruence is observed showing contralateral activation to the modeled hand. In third-person viewpoint, specular or spatial congruence is seen with parietal activation ipsilateral to the modeled hand (Shmuelof and Zohary, 2008; Vingerhoets et al., 2012b).

### INTEGRATION OF REACHING AND GRASPING

Although the transport and grip components of a transitive gesture can be separated conceptually, in everyday life they present as a single fluid action. Much of the research thus far has strived toward the study of one single component as if reaching and grasping were completely independent. As shown above, supporting evidence from neuropsychology and neuroimaging indeed points to a aIPS – vPM circuit (also termed the dorsolateral circuit) relevant for grasping that can be distinguished from a POTZ (SPOC)/mIPS – dPM circuit (the dorsomedial circuit) underlying reaching (**Figure 1C**). Novel paradigms combining reaching and grasping uncovered brain regions (supplementary motor area, SMA and dPM) that seem to be active during both components and may be relevant for the coordination of reach and grasp (Cavina-Pratesi et al., 2010). In addition and adding to the observations reported above (Tunik et al., 2008b), recent evidence suggests that the aIPS centered dorsolateral circuit and the superior POS centered dorsomedial network appear to specify the same grasping parameters but are temporarily dependent on each other, and thus seem to be organized in a hierarchical manner (Verhagen et al., 2013).

## USING

### DEFINITION

Transitive movements are performed with a purpose. The purpose of the interaction dictates how we will grasp and manipulate an object. This is very obvious when we interact with tool objects. Manipulating a pair of scissors to cut a piece of paper for example, is quite different from the gestures required to move the scissors from the desktop to the drawer. But goal-directed differences are also observed when we interact with objects that have no particular function; the way I will pick up a stick to throw it away for my dog to fetch is different from the movements I use to move the stick out of the way (Ansuini et al., 2006, 2008). Using an object for a particular purpose requires the generation of an action plan. Usually, this plan is already present during the reach and grasp components of the transitive action. Top-down motor planning in grasping is nicely demonstrated by the end-state comfort effect, the tendency of people to adaptively structure their initial grasp in order to end up with a comfortable posture for the intended action, even when this necessitates them to use an awkward grasp at the start of the movement (Rosenbaum et al., 1992, 1996). When grasping a cup that is upside down, we would use a different grip when we want to pour tea in it compared to placing it in the dishwasher. Behavioral research has provided support for a left hemisphere dominance in the motor planning of end-state comfort effects in right and left handers (Janssen et al., 2011). It remains a matter of debate whether the planning of reach and grasp actions for object use versus object transport are guided by different mechanisms (Osiurak et al., 2008). In addition to reach-and-grasp planning, we also must recall and apply the appropriate object-related movements to achieve the planned goal. Again, the different components of the transitive action, reaching, grasping, and using, are closely intertwined, and difficult to separate in natural action.

Purposeful behavior with objects can be assessed in a variety of ways, of which the *actual use* of tools appears to be the most ecological method. But other approaches have been fruitful too. Clinical work revealed that *pantomiming* tool use is a more sensitive method to elicit symptoms of apraxia, as patients are unable to rely on the physical properties of the tool that may afford tool-related gestures (Randerath et al., 2011)*. Imaging* the use of tools has been applied to make abstraction of the actual movements and investigate the neural and behavioral correlates of motor imagery. Finally, researchers have also investigated the *receptive*, rather than *productive* aspects of tool use by having participants observe actual or pantomimed tool-related behavior performed by others. Motor imagery and action observation are sometimes referred to as action simulation states or S-states, because they appear to be based on the activation of the brain’s motor system, yet in contrast to actual or pantomimed movements, they require no execution of the motor action (Jeannerod, 2001). We will offer an overview of the most relevant findings for each of these tasks below.

### PRODUCTIVE PARIETAL RESPONSES OF TRANSITIVE GESTURES

#### Actual use of objects and tools

***Neuropsychological research.*** Misuse of everyday tools and objects is one of the three categories of symptoms that qualify for the diagnosis of apraxia {the other two being dysfunctions in the imitation of gestures and the production of communicative gestures [symbolic gestures (also called emblems) or pantomimes] respectively Goldenberg, 2008, 2009}. Apraxia occurs predominantly following left brain lesions and affects both sides of the body, not just the (often hemiplegic) contralesional side. Patients with apraxia may present with multiple or just one of the core symptoms indicating that apraxia is not a unitary disorder and that the different symptoms rely on a (partially) different neural representation. Clinical neuropsychology has traditionally associated limb apraxia with left parietal dysfunction. In particular the left IPL and IPS region are assumed to store knowledge about hand and finger postures/movements required for the use of tools (Sirigu et al., 1995; Haaland et al., 2000; Buxbaum et al., 2003, 2007), but also see (Schnider et al., 1997). Although Goldenberg (2009) claimed that no clear relation between defective actual tool use and left parietal lesions has been established, other than a number of case studies, his voxel-wise lesion-function study revealed selective impairment on certain tool tasks following parietal damage (Goldenberg and Spatt, 2009). In this study Goldenberg and Spatt (2009) investigated 38 patients with left brain damage on semantic tool knowledge, mechanical problem solving, and use of familiar tools and objects. Parietal lesions, in particular of the IPL and SMG, interfered with the latter two tasks, but not with semantic tool knowledge. The authors concluded that the parietal lobe’s role concerns general principles of tool use and comprehension of mechanical interactions, rather than prototypical tool use gestures or the selection of grip formations (Goldenberg and Spatt, 2009). A related observation of a dissociation between functional object knowledge (action semantics) and mechanical problem solving skills has been voiced earlier (Hodges et al., 1999). Patients with semantic dementia and temporal atrophy showed impaired object identification and functional semantics and displayed markedly impaired use of familiar objects, yet retained mechanical problem solving ability as demonstrated in a novel tool task and the correct use of familiar objects with obvious structure-function relationships (Hodges et al., 2000). In contrast, a patient with corticobasal degeneration and biparietal atrophy demonstrated impaired mechanical problem solving and common tool use despite near normal semantic knowledge about the tool’s function (Hodges et al., 1999). It appeared to the authors that object-specific conceptual knowledge is crucial for object use, and may be supplemented to some degree by sensory input of object affordances into a parietal “how” system that may trigger mechanical reasoning and the correct use of (some) objects (Hodges et al., 1999, 2000). Later research challenged this view by presenting two patients with degraded semantic knowledge (including functional object knowledge), who showed preserved object use over a two-year follow-up (Negri et al., 2007). The existence of a separate representation of semantic *and* kinematic/motor knowledge of functional object use in the brain thus remains to be elucidated.

***Neuroimaging research.*** Given the limitations for tool interaction in the scanner environment, only a handful of fMRI studies examined actual tool use in humans. Their paradigms compared real tool manipulation against pantomimed or imagined use, or both. In general, the tasks produced widespread activation in parietal, posterior temporal, frontal, and subcortical regions. We will again focus on specific task differences within the parietal region. One study investigated the actual, pantomimed, and imagined right hand use of chop sticks (Imazu et al., 2007). Compared to the pantomimed performance, actual chop stick use showed increased parietal activation in the left PoCG and right IPL (BA 40). Another study compared the actual use of 16 common tools or their imagined use with a control condition without mental task (Higuchi et al., 2007). In the latter two conditions participants were allowed to hold the tools. Actual use revealed unique activity in the left postcentral gyrus and shared activity with the imagery task in left pIPS compared to the control task. A third study compared pantomimed and actual use of 32 familiar objects during a presentation phase, a preparation phase, and an execution phase during which they were either handed the tool for actual use, or were required to pantomime its use (Hermsdörfer et al., 2007). During the execution phase, actual tool use revealed increased activation in left PoCG, and bilateral SPL and IPL (BA 40).

**FIGURE 3:**
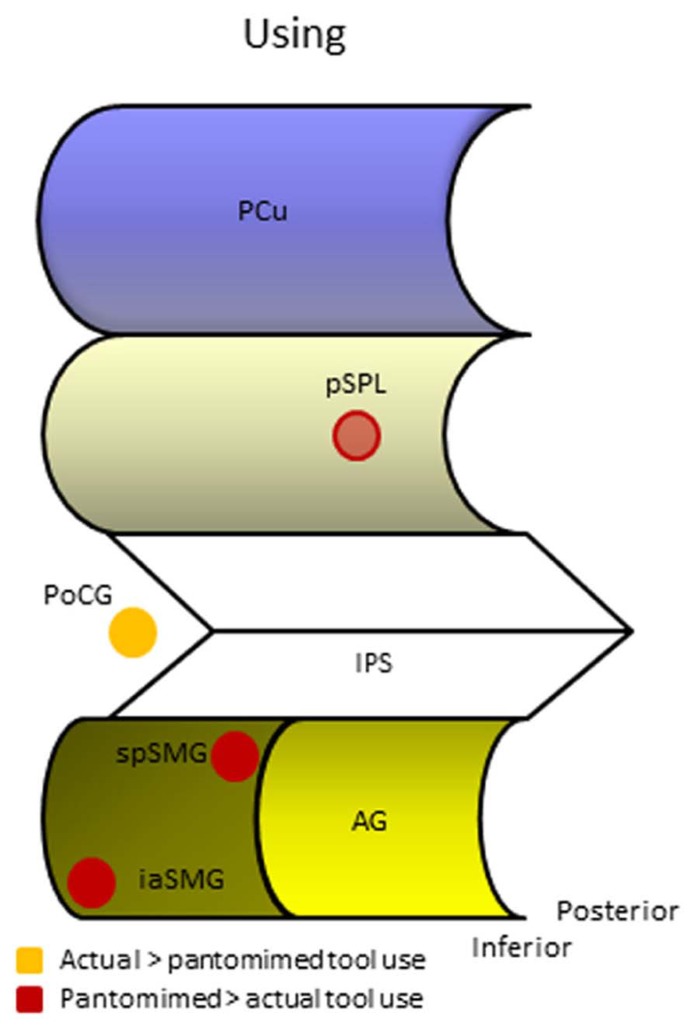
**Human parietal foci responsive during different manifestations of tool use.** AG, angular gyrus; iaSMG, inferior anterior supramarginal gyrus; IPS, intraparietal sulcus; PCu, precuneus; PoCG, postcentral gyrus; pSPL, posterior superior parietal lobule; spSMG, superior posterior supramarginal gyrus.

As expected, all studies report increased activation during actual tool use over the left primary somatosensory region (PoCG, **Figure 3**). Modulation of several bilateral posterior parietal regions is also reported, but there is little consensus regarding a specific location which is probably due to substantial methodological differences between studies. The additional somatosensory modulation during real tool manipulation suggests that the physical demands of the object may serve as cues during actual performance and might explain why apraxic patients perform typically better during actual tool use than during pantomimed tool use (Laimgruber et al., 2005; Hermsdörfer et al., 2006; Randerath et al., 2011).

Another interesting approach compared the neural activation in healthy right handers during the manipulation of a small object with a pair of tongs or with the fingers (Inoue et al., 2001). The PET study revealed that when volunteers used their dominant hand, the left aIPS was activated similarly in the tool and fingers condition, but in the tool condition an additional region in the ipsilateral (right) posterior IPL/IPS became active. The authors interpreted this region to be involved in the integration of visuosomatosensory information during the use of a tool as required during the incorporation of an external object into the body schema (Inoue et al., 2001; Maravita and Iriki, 2004).

#### Pantomimed use of objects and tools

***Neuropsychological research.*** The crucial difference between pantomimed versus actual use of a tool is that the former has to be mentally elaborated and stored in the absence of an external image of the object and the hand acting on it. In other words, it is a creative process that cannot rely on the physical cues provided by the action scene. Movement kinematics of actual versus pantomimed prehensile actions demonstrated qualitative differences between both tasks in apraxic patients *and* healthy controls (Laimgruber et al., 2005; Hermsdörfer et al., 2006). Apparently, patients with apraxia experience difficulties with the absence of the mechanical affordances and constraints of real tools and objects as their performance during naturalistic execution is often superior to their pantomiming of similar actions (Goldenberg and Hagmann, 1998; Buxbaum et al., 2000; Westwood et al., 2001; Goldenberg et al., 2004). Similar to actual tool use, the involvement of parietal lesions with deficits in pantomiming meaningful gestures on verbal command is mainly supported by single case observations, but not corroborated by lesion studies (Goldenberg et al., 2003, 2007; Goldenberg, 2009). The lack of a clear relationship between left parietal lesions and tool use pantomiming, and a somewhat more convincing (though not absolute) relation of parietal damage with deficits in actual tool use and imitation of meaningless gestures, had led Goldenberg to the question of what the latter tasks have in common. Goldenberg proposes that tool use and imitation of meaningless gestures rely on the categorical apprehension of spatial relationships between body parts, tools, and objects. Rather than a repository for the representation of motor acts, the parietal lobe acts to spatially configure multiple (parts of) objects, that may be body parts, external objects, or both (Goldenberg, 2009).

***Neuroimaging research.*** In contrast to the limited evidence of a relation between parietal lesions and pantomime dysfunction, most (if not all) of the neuroimaging studies using pantomime paradigms reported robust posterior parietal activation in their healthy subjects. One of the first studies compared tool use pantomimes versus a non-symbolic gesture sequence and found predominant left IPS activation (Moll et al., 2000). Choi et al. (2001) compared left and right hand tool pantomiming against a motor control task and demonstrated dominant left parietal activation with either effector. Tool pantomiming resulted in activation of the SPL (BA 7) and SMG (BA 40), with stronger activation in the former (Choi et al., 2001). Rumiati et al. (2004) used PET in a paradigm that controlled for perceptual, semantic, and sensorimotor aspects to reveal that skilled pantomimes elicited parietal activation in two left IPL foci. The more dorsal and posterior one is particularly responsive to pantomiming triggered by object stimuli, whereas their more ventral IPL focus was also active during imitation of pantomimes and was taken by the authors to be associated with tool grasping (Rumiati et al., 2004). Ordinary tool pantomimes and body-part-as-object gestures showed left SPL (BA 7) and SMG (BA 40) activity irrespective of the hand used in the study of Ohgami et al. (2004). Body-part-as-object gestures additionally activated the right SMG (Ohgami et al., 2004). Johnson-Frey et al. (2005) compared the planning and execution of tool use gestures with either hand against a movement control task. For either limb planning tool use pantomimes activated two left parietal foci in the IPL, one more anterior and inferior in SMG, another more superior and posterior in SMG extending to AG (Johnson-Frey et al., 2005). The authors noted that the anterior focus did not match with the putative human AIP coordinates derived from human grasping studies, and suggested that representations of tool manipulation are stored in a separate region, that is near to, but not identical with the area involved in computing sensorimotor transformations during grasping. Johnson-Frey et al. (2005) further hypothesize that their posterior parietal SMG/AG site is involved in the representation of motor programs for acquired tool use skills. Imazu et al. (2007) found increased activity in the left IPL (BA 40) during pantomimed compared to actual chop stick use. ROI-analysis over the left parietal region also revealed an IPL focus of the pantomime versus actual use contrast in the Hermsdörfer et al. (2007) and suggested that this region may be necessary for pantomiming, but unnecessary when sensorimotor feedback is available (Hermsdörfer et al., 2007). Kroliczak and Frey (2009) investigated tool pantomime versus a linguistic control task. Two left IPL foci appeared to be independent of the hand used to prepare the pantomime. A first was positioned dorsal and posterior in the IPS, the second more ventral and anterior in the SMG. Three recent studies by Vingerhoets et al. (2011, 2012a, 2013a) investigated pantomiming of familiar tools to explore action semantics and the effects of handedness and atypical language lateralization respectively. In a first study, a distinction was made between execution and planning of tool pantomimes controlled for non tool transitive movements. Execution of familiar tool pantomimes bilaterally modulated SPL and two foci within IPL. The more dorsal focus lies in the superior part of SMG, whereas the ventral focus is located in the part of the SMG that descends into the lateral fissure (Vingerhoets et al., 2011). A hand-independent paradigm comparing tool pantomiming versus control gestures was used in two other studies that evaluated neural lateralization effects due to hand preference and language dominance respectively. Tool pantomimes elicited robust left parietal activation regardless of handedness and hand-effector, although lateralization in the parietal region was stronger in right handers compared to left handers (Vingerhoets et al., 2012a). Typical (left) language dominant volunteers exhibited activation in the left PoCG, PCu, and two SMG foci that appeared switched to homologous regions in the right hemispheres in participants with atypical (right) language dominance (Vingerhoets et al., 2013a).

What most of the studies investigating tool pantomiming in normal volunteers seem to have in common is the activation of one or both of two foci in the left SMG that appear to differ along a superior/inferior and anterior/posterior dimension (**Figure 3**). A summary of the peak coordinates grouped along these dimensions is provided in **Table 1**. We only listed peak voxel coordinates if provided by the studies, and if in the IPL (the SMG coordinate reported by Choi et al., 2001 for example is not even near SMG). MNI coordinates were Lancaster-transformed to Talairach coordinates if necessary and vice versa (Lancaster et al., 2007). Coordinates were ordered along the *Z*-axis with more superior peak voxels on top. If you now look at the *Y*-axis Talairach coordinates, you see that in the upper part of the table most *Y*-coordinates are close to 45 (indicated in blue), whereas in the lower part they are more close to 35 (indicated in green). Two outliers, indicated in red, were not used in calculating the mean coordinates of both foci. The more superior and posterior focus of the two (-42, -46, 48, Talairach coordinate) is positioned on the convex portion of the posterior part of the SMG. Its activation has been attributed to the triggering of object-related action schemata in humans (Rumiati et al., 2004), the representation of motor programs for acquired tool use skills (Johnson-Frey et al., 2005), and the production of object manipulation without sensory feedback (Imazu et al., 2007). The more inferior and anterior focus (-53, -33, 31, Talairach coordinate) is located on the ventral part of the SMG were the gyrus descends in the lateral fissure. This focus has been related to tool use and grasping in particular (Rumiati et al., 2004), and appears active during both planned and executed gestures (Johnson-Frey et al., 2005; Hermsdörfer et al., 2007). Despite its anterior position, this anterior SMG focus is not similar to the aIPS, the prototypical region underlying grasp formation (Tunik et al., 2007). Tunik et al. (2007) listed 22 coordinates of human aIPS in grasping studies and calculated the mean coordinates for the left and right hemisphere (-40, -39, 43 on the left, indicated in brown in **Table 1**, and 41, -40, 45 on the right). Clearly, the anterior SMG focus found in tool pantomime studies lies inferior and lateral to this region. In addition, tool pantomime neuroimaging studies show robust left lateralized activation, irrespective of the hand used, which is fundamentally different from the more robust contralateral aIPS activation that depends on the hand performing the grasping movement. Taken together, these results suggest that tool use pantomiming elicits activation in left hemispheric anterior and posterior SMG foci that can be distinguished from the prototypical grasp formation aIPS locus. Given the diverse interpretations, it is difficult to speculate on the role of these different foci. The left anterior SMG focus may seem to be particularly tuned to the grasping of familiar objects (compared to unknown objects or shapes), but this was not confirmed in a study that explicitly tested this assumption and in fact revealed more posterior SMG activation for such a contrast (Vingerhoets et al., 2009a). The only contrast that demonstrated modulation of the anterior SMG in the latter study was when participants had to imagine displacing familiar versus unfamiliar tools (-58, -23, 33, Talairach coordinate), suggesting that this region may indeed be object-specific, but not particularly tuned to functional prehension (Vingerhoets et al., 2009a). An alternative explanation may be found in a transcranial magnetic stimulation (TMS) study during planning of grasp actions (Tunik et al., 2008a). These researchers administered TMS over left SMG during goal specific grasping movements toward a familiar object (a cup placed upside down) with the intent to use or to move the object. Although no specific site coordinates were provided, the SMG focus illustrated in their figure lies inferior and anterior to the aIPS focus (another TMS target in their study) and thus seems to coincide with a more anterior SMG location. Stimulation to SMG (but not aIPS) during the planning phase of the action significantly delayed the goal-oriented actions, although the execution of arbitrary stimulus-response mappings was not affected. Based on these and previous data, Tunik et al. (2008a) argued that SMG may be involved in goal-oriented formation of plans and selection of actions (planning of actions), whereas aIPS may be responsible for monitoring the fit between hand-object interactions and its intended outcome (guidance of actions). More recently, and partially based on diffusion tensor imaging to identify connections between tool-relevant brain regions, it was proposed that the anterior SMG is responsible for the integration of non-spatial and semantic information to generate a gesture plan as it appears to show a strong and almost completely left lateralized connection with the posterior middle temporal gyrus, a region that is considered to be a repository of semantic information (Ramayya et al., 2010). Taken together, the anterior SMG region may be involved in goal-specific movement planning toward tool-like objects, but more specific research is needed to corroborate this idea. There is more consensus on the role of the posterior SMG subserving functional motor schemata for familiar objects. The imagined use of familiar tools repeatedly demonstrated increased activation in the left posterior SMG region when compared to imagined displacement of tools (familiar and unfamiliar) or shapes (Vingerhoets et al., 2009a).

**Table 1:** Inferior parietal lobule foci reported in pantomime studies.

Study	Contrast	Side	Location	*X* (tal)	*Y* (tal)	*Z* (tal)	*X* (mni)	*Y* (mni)	*Z* (mni)
Johnson-Frey et al. (2005)	Pantomime tool (plan) RH > control movement RH	Left	pSMG	-38	-52	56	-39	-48	64
Vingerhoets et al. (2011)	Pantomime tool(exec) > pantomime tool (plan)	Left	IPL (40)	-30	-38	54	-30	-33	60
Johnson-Frey et al. (2005)	Pantomime tool (plan) LH > control movement LH	Left	pSMG	-42	-52	50	-43	-49	57
Kroliczak and Frey (2009)	Pantomime tool(plan) > linguistic control LH	Left	IPL	-41	-51	47	-42	-48	54
Imazu et al. (2007)	Imagined use > actual use	Left	IPL (40)	-58	-44	46	-61	-41	52
Rumiati et al. (2004)	Pantomime skilled tool use	Left	IPL (40)	-52	-44	46	-54	-41	52
Johnson-Frey et al. (2005)	Pantomime tool (plan) LH > control movement LH	Left	IPS	-30	-42	45	-31	-39	50
Kroliczak and Frey (2009)	Pantomime tool(plan) > linguistic control RH	Left	IPL	-44	-49	44	-46	-46	50
Imazu et al. (2007)	Pantomime > actual use	Left	IPL (40)	-52	-44	44	-54	-41	50
Johnson-Frey et al. (2005)	Pantomime tool (plan) LH > control movement LH	Left	aSMG	-59	-25	44	-62	-21	48
Vingerhoets et al. (2013a)	Pantomime tool (exec) > transit control pantomime	Left	IPL (40)	-33	-45	43	-34	-42	48
Vingerhoets et al. (2013a)	Pantomime tool (exec) > transit control pantomime	Left	IPL (40)	-41	-34	42	-43	-31	46
Hermsdörfer et al. (2007)	Pantomime > actual use (ROI)	Left	IPL (40)	-29	-62	41	-30	-60	48
Johnson-Frey et al. (2005)	Pantomime tool (plan) RH > control movement RH	Left	aSMG	-62	-28	35	-65	-25	38
Johnson-Frey et al. (2005)	Pantomime tool (plan) RH > control movement RH	Left	aSMG	-50	-29	33	-52	-26	36
Kroliczak and Frey (2009)	Pantomime tool(plan) > linguistic control LH	Left	IPL	-50	-40	30	-52	-38	34
Rumiati et al. (2004)	Pantomime skilled tool use	Left	IPL (40)	-58	-32	30	-61	-30	33
Kroliczak and Frey (2009)	Pantomime tool(plan) > linguistic control RH	Left	IPL	-55	-38	27	-58	-36	30
Vingerhoets et al. (2011)	Pantomime tool(exec) > pantomime tool (plan)	Left	IPL (40)	-57	-33	23	-60	-31	25
	A superior posterior SMG focus (spSMG mean coordinate)			-**42**	-**46**	**48**	-**43**	-**43**	**54**
	An inferior anterior SMG focus (iaSMG mean coordinate)			-**53**	-**33**	**31**	-**56**	-**31**	**34**
Tunik et al. (2007)	aIPS (mean of 22 human aIPS coordinates)			-**40**	-**39**	**43**	-**41**	-**36**	**48**

*Coordinated are provided according to Talairach and MNI coordinate systems. Green coordinates are more akin to an inferior anterior SMG focus, whereas blue coordinates refer to a superior posterior SMG location. Coordinates with red markings are not used in averaging. Brown coordinates refer to an averaged aIPS coordinate. Bold coordinates reflect mean coordinates of multiple foci.*

Why is this robust left IPL activation during tool use pantomime in healthy participants not reflected in a clear cut relation between parietal lesions and pantomime dysfunction? Goldenberg (2009) suggests that the unusual position and awkward visual and spatial context of the participant during imaging studies may give rise to additional spatial demands that induce this parietal activation. It can be argued that this possible confound would not hold for planned or imagined pantomimes, and that the latter tasks nevertheless reveal significant left IPL activity. An alternative explanation may be that a left parietal lesion often does not suffice to elicit deficient pantomiming. Although the left PPC may be involved in the initiation and control of motor schemata for pantomiming the use of familiar objects, the existence of multiple and potentially redundant left parietal foci, and the modulation of right parietal regions during the pantomiming of familiar and unfamiliar tools hints at the availability of compensatory mechanisms (Vingerhoets et al., 2011). Additional frontal or white matter damage may be required to disrupt the execution of the (sub-optimal) pantomime plan.

#### Imagined use of objects and tools

Imagined tool use is a strategy used in some neuroimaging studies to avoid possible noise of overt movements in the magnet or to explore the neural correlates of mental imagery. The drawback of imagined gestures is of course a lack of performance and compliance data, although the temporal coupling between imagined and executed movements can be used for a timed estimation of the imagery performance (Vingerhoets et al., 2009a). In addition, it is unclear how “imagined tool use” differs from “planned pantomime,” an approach that introduces a delay period between stimulus presentation and the execution command (Johnson-Frey et al., 2005; Kroliczak and Frey, 2009). During the delay period participants are required to prepare the instructed gesture, but it is unclear whether this required keeping the task active in working memory or to imagine its execution. Here, I will focus on studies that explicitly requested imagined tool use.

Imazu et al. (2007) found increased activity in the left IPL (BA 40) during imagined and executed pantomimes compared to actual chop stick use. They suggested that this left IPL focus was involved in the explicit retrieval and production of grasping and manipulation of objects without sensory feedback. Moll et al. (2000) reported IPS patterns of activation during imagined tool-use performance (versus an imagined control motor task) that were identical to those during a similar pantomimed contrast. Vingerhoets et al. (2009a) compared the parietal activation during imagined use versus imagined displacement of the same tools and uncovered activation in left SPL extending to mIPS and aIPS. Unfortunately this study only compared different manipulations of target objects and tasks, it did not include a condition with executed pantomimes. Interestingly, a very strict conjunction analysis aimed to reveal voxels that are activated while using a familiar tool (in imagination) while correcting for differences in object qualities or non-functional aspects of reach-and-grasp movements, detected significant activity on the convex border of the left SPL/SMG in a region that is very close to the posterior SMG focus described in the pantomime section (Vingerhoets et al., 2009a). Again, this finding confirms the involvement of the posterior SMG region for the representation of motor schemata for the functional use of tools. The few studies on imagined (rather than executed) pantomimes seem to indicate that imaging tool use produces activation in the same regions that are active during the real pantomiming of tool use, confirming the close neural match between motor imagery and executed movement (Jeannerod, 2001; Kosslyn et al., 2001).

### RECEPTIVE PARIETAL RESPONSES OF TRANSITIVE GESTURES

#### Observed actual use of objects and tools

Viewing tool objects facilitates motor responses that are compatible with its manipulation (Humphreys, 2001). The object is believed to possess affordances, properties that are relevant for its use and potentiate associated motor actions (Gibson, 1979). Effects of action priming or motor affordances have been described in particular for physical object properties such as its size or spatial orientation (Tucker and Ellis, 1998, 2001; Phillips and Ward, 2002; Grezes et al., 2003b; Vingerhoets et al., 2009b). As tools, in contrast to most other classes of objects, are able to activate cortical areas associated with motor functions, action priming is believed to result from the neural activation elicited by the tool object that partially overlaps with regions involved with actual tool use (Decety et al., 1997; Chao and Martin, 2000; Grezes and Decety, 2002; Grezes et al., 2003b; Creem-Regehr and Lee, 2005; Lewis, 2006; Vingerhoets, 2008). Here, we will focus on the neural correlates of observed tool use, rather than of static images of tools. A meta-analysis of the neural patterns of execution, simulation of execution, and observed execution of actions revealed clear overlap in SMG and SPL, among other extraparietal motor-related regions (Grezes and Decety, 2001). Other studies reported strong activation of the aIPS and IPL during the observation of transitive actions (Buccino et al., 2001; Manthey et al., 2003), or overlap in the left IPL in a conjunction analysis of observed and executed transitive actions (Hetu et al., 2011). Peeters et al. (2009) scanned human volunteers, untrained monkeys, and two monkeys trained to use tools, while they observed hand actions and actions performed using simple tools. During tool use observations, human participants exhibited specific activation of a rostral region in left IPL (aSMG) that was not observed in the untrained and trained monkeys. The authors claim that this uniquely human region underlies a specific way of understanding tool actions in terms of causal relationships between the intended use of the tool and the results obtained by its use, and represents a fundamental evolutionary leap enlarging the motor repertoire of humans (Peeters et al., 2009). Interestingly, the aSMG coordinate reported in this comparative fMRI study (-52, -26, 34, MNI coordinate) is very similar to the mean iaSMG coordinate reported in the tool use pantomime section and **Table 1** (-56, -31, 34, MNI coordinate).

#### Observed pantomimed use of objects and tools

Halsband et al. (2001) showed patients with left or right parietal or premotor lesions video clips of familiar pantomimed gestures. They were asked to recognize the gestures and subsequently imitate them from memory. The patients showed little problems with gesture comprehension, but the left parietal volunteers were most severely disturbed on imitation performance, especially with gestures on their own body (combing one’s hair) rather than with an external object (hammering a nail). In a related study, healthy volunteers observed similar sets of pantomimes while undergoing fMRI (Lotze et al., 2006). aIPS was activated in body-referred and isolated hand pantomimes, whereas left inferior SMG and AG showed a significantly increased response to body-referred pantomimes compared to an isolated hand pantomiming an external object.

## CONCLUDING REMARKS

### PARIETAL REGIONS IMPLICATED IN REACHING, GRASPING, AND TOOL USE

*Reaching* involves the transportation of the limb effector toward the target, a task that is usually performed under visual guidance and thus requires the integration of visual and proprioceptive coordinates in a network that is able to respond flexibly to changing target positions and effector facilities. Human parietal areas associated with this ability include pIPS and superior PPC areas. pIPS regions appear to deal with the correspondence of visual and motor coordinates, whereas more rostral superior SPL/PCu areas seem to provide input regarding target related proprioceptive information. Both regions are believed to interact during reaches and share specifics of grasps.

*Grasping* is regarded as the act that completes the transitive movement, the merging of hand and object. As in reaching, it is likely to be guided visually and also requires close interplay with proprioceptive information. As a result, there is a substantial overlap in parietal regions subserving reaching and grasping tasks, especially along the IPS. Grasp specific areas include the aIPS and probably also cIPS. The former is a well-established grasping region involved in the perception and execution of prehensile hand configurations and very similar in function to monkey AIP, although in humans its function also appears to encompass goal-directed action planning. cIPS is believed to play a role in prehension-related texture analysis. As in reaching, more superior and medial (SPL) areas are shown to respond when on-line adjustments of the grasping movement seem necessary.

In contrast to reaching and grasping that seem present at birth, the *functional use* of objects and tools requires the recall of learned object interactions. Tool manipulation knowledge can be demonstrated in a variety of ways. In general, neuropsychological and neuroimaging studies agree on a strong left hemispheric lateralization for praxis, although they don’t always agree on the key role of the PPC. Compared to reaching and grasping, the left hemispheric dominance of praxis, regardless of which hand performs the task, underscores the more conceptual level of the mental operations involved. When contrasted to simple motor control tasks, tool use paradigms demonstrate widespread activation along the IPS and adjacent areas. But given its many possible task comparisons tool use paradigms usually explore more fine-grained task differences concerning stimulus type, movement goal, effector choice, assessment method, etc. Subtraction of similar tool use activation patterns reflecting subtle task differences offer detail on the functional role of particular cortical areas, but also filter away most of the basic prehensile PPC activation, and may make us unobservant of its key role in every goal-directed transitive action. This being said, comparison of different tool use tasks reveals that actual tool use is accompanied by activation of the sensorimotor cortex and this might help apraxic patients in the recall of the appropriate tool use gestures. When this proprioceptive feedback is absent, as during tool use pantomiming or tool use imagery, individuals are more reliant on memorized tool interactions, and this appears to elicit neural responses in the IPL. Possibly, multiple IPL foci exist, mediating different types of information of learned transitive movements and interactions. Similar to the reaching and grasping of objects, tool use also seems associated with SPL activation, although it may not be the same SPL regions that contribute to each of the action components. The core regions underlying reach and grasp gestures, however, are organized along the IPS, whereas the core regions subserving functional object use activate the phylogenetic new inferior parietal cortex.

### THE PUTATIVE ROLE OF THE POSTERIOR PARIETAL REGION

Traditionally, the parietal cortex is considered as a major component of a dorsal visual pathway (occipital-parietal route) that encodes spatial location (“where” an object is) and can be differentiated from a ventral visual pathway (occipital-temporal route) responsible for object identification (“what” an object is; Ungerleider and Mishkin, 1982). Later, Goodale and Milner (1992) re-interpreted the functional role of the dorsal visual stream from “where” to “how,” taking into account its prominent role in the control of skilled motor action.

Both the reaching and grasping literature, and the research on tool use – which seem to have evolved as two relatively separate lines of research – have proposed further subdivisions of the dorsal stream. Based on animal research and clinical data a differentiation of the dorsal stream was proposed into a dorso-dorsal part important for the online control of the transitive action and a ventro-dorsal stream involved with action organization (Tanne-Gariepy et al., 2002; Rizzolatti and Matelli, 2003). In the monkey brain, a dorso-dorsal stream originates from an extrastriate visual node V6, and connects with areas V6A and the medial intraparietal area (MIP) in the medial bank of the IPS (SPL), which is closely linked to the somatosensory system. Its major functional role is described as important for the “on-line” control of action. A ventro-dorsal stream stems from another extrastriate node MT (middle temporal), and connects to the IPL and medial superior temporal area (MST). In addition to action organization, the ventro-dorsal stream is hypothesized to play a role in object awareness, control of hand posture, and action understanding (Rizzolatti and Matelli, 2003). Based on neuropsychological evidence and functional imaging data, it is suggested that a comparable segregation might exist in humans, one for acting on and another for acting with objects, and that hand-object interactions follow different streams dependent on the goal to be achieved (Johnson and Grafton, 2003; Johnson-Frey, 2004; Buxbaum et al., 2006, 2007; Daprati and Sirigu, 2006; Vingerhoets et al., 2009a). If an object is to be moved (acting on), visual information regarding the object’s intrinsic (shape and size) and extrinsic (orientation and location) qualities will guide the movement’s reach trajectory and grasp aperture. Conceptual knowledge about the target is not required and the movement is guided by an IPS/SPL network. If we wish to use the object (acting with), stored knowledge about its functional properties is required and is integrated with the perceptual affordances of the “on-line” pathway. The conceptual input is believed to rely on IPL structures and guides a functional grasp and purposeful movement with the object.

Similarly, reach and grasp research proposed a distinction between dorsomedial (transport) and dorsolateral (grip) substreams within the parieto-frontal cortex (Grol et al., 2007; Cavina-Pratesi et al., 2010; Vesia and Crawford, 2012). Dorsomedial parietal regions include SPOC, whereas dorsolateral regions include aIPS, although a significant crosstalk between both substreams is expected (Grol et al., 2007).

Both the tool use and the reach and grasp lines of research suggest a division of the dorsal stream in two functionally different substreams based on empirical findings within each research tradition. Although there are clear similarities between the proposals, there are also differences, with the transport/grip distinction focusing on SPL/IPS regions and their frontal projections and the more transitive “use” literature concentrating on the separate contribution of perceptual versus semantic information in action control and the importance of IPL for the latter. A possible integration of both views would be to consider a reach-and-grasp movement as the backbone of every transitive gesture. Such an act requires the integration of visual and somatosensory information that in primates appears to be organized mainly along the IPS in a mosaic of areas that have graded input to the different components (reach or grasp), modalities (visual, somatosensory), and effectors (hand, arm, eye) that contribute to the reach and grasp movement. Its complexity reflects the primate’s ability of performing complex transitive actions associated with independent bilateral control over hands with opposable thumb*s* and its cortical network occupies a bilateral IPS territory that bridges incoming visual and somatosensory input. At the same time, the core IPS-centered reach-and-grasp process is supported (and if necessary corrected or adapted) by two different sources of information. The first is predominantly sensory and perceptual in nature and subserves corrections due to more demanding visuo-spatial/tactile-proprioceptive matches. It contributes to the on-line control of the transitive action and is mainly performed in dorsomedial PPC, in particular SPL. The second source of information that contributes to the reach-and-grasp process is more semantic in nature as it relies on functional knowledge about the object, previous experience with the associated actions, and probably also on acquired insight in mechanical relations. The IPL may not necessarily be the repository of all of this knowledge, but it somehow controls the way in which action semantics influences the transitive gesture. This source of information and its influence on the transport/grip action is clearly lateralized, usually to the left. It is especially well-developed in humans and may constitute a major difference with non-human primate transitive movements. For a proper understanding of the role of each of these processes, it is important to note that a reach and grasp action is not completed once the target object is held. Subsequent object manipulation requires continued adaptations of visuospatial coordinates (transport) and hand posture (grip) in order to carry out the desired transitive movement. Depending on the type and phase of the transitive action, differential input from both information sources is continuously necessary to steer transport and grip components adaptively in a given situation and environment.

### EFFECTOR-SPECIFIC, SIDE-SPECIFIC, AND ACTION-SPECIFIC PARIETAL MAPPING

In the paragraphs on reaching and grasping, we already pointed to the lateralized effects of hemi-field presentation (reaching to targets in right or left hemi-space produces more robust contralateral pIPS activation) and effector performance (reaching with the right or left effector evokes more robust contralateral activation in anterior IPS; Blangero et al., 2009). Many findings also have led to the view that PPC is organized in an effector-specific manner, with different subregions mediating movements for hand, arm, and eye respectively. What remains uncertain is the degree of effector and computational specificity of these regions in particular in the human PPC (Vesia and Crawford, 2012). In a review on reach function these authors investigated effector specificity of reach versus saccades and reach versus grasp to come to the conclusion that there is empirical evidence for the existence of effector specificity *and* for a substantial overlap. Increases in the spatial resolution of current neuroimaging techniques appears required to shed more light on the effector specificity of human reach and grasp movements.

In tool use research lateralized effector-specificity seems to be of lesser importance. The side of the hand performing the tool manipulation has little effect on the strong leftward parietal activation, and even left handers show a clear left dominant praxis network (Vingerhoets et al., 2012a). The type of effector used however does seem to elicit some effector-specific mapping in PPC. Buccino et al. (2001) had volunteers observe transitive and intransitive gestures performed by hand, leg, and mouth and their results demonstrated effector specificity in the PPC of both hemispheres. Later research also pointed to the existence of PPC regions that showed overlapping activation during similar actions observed by different effectors, suggesting a form of action mapping that is independent of the effector performing the task (Jastorff et al., 2010; Heed et al., 2011; Lorey et al., 2013). As this research mainly focused on S-states (observation and imagery) and not on actual effector performance, and effector and action specificity appear to depend on the type of S-state applied (Lorey et al., 2013), further research appears necessary. The discovery of action-specific mapping is an intriguing finding that begs the question along which dimensions transitive action-specificity is organized. Given its association with the functional meaning of transitive actions, action mapping regions may be expected to be found in an IPL location and this indeed seems to be the case (Jastorff et al., 2010; Lorey et al., 2013).

## Conflict of Interest Statement

The author declares that the research was conducted in the absence of any commercial or financial relationships that could be construed as a potential conflict of interest.

## Footnotes

1 Although I realize that the words “object” and “tool” do not convey exactly the same meaning, I will use them interchangeably in the manuscript. In both cases, I consider them as external objects that serve a functional purpose in the transitive action, such as a scissors applied to cut a piece of paper or a pebble thrown to demonstrate my aiming skills.

2 Although one can also reach to a location or to a person, I will focus on reaching toward an external object. Pointing may also encompass a transportation of the hand, but is not considered here.
